# A Comparative Study of Peripheral Immune Responses to *Taenia solium* in Individuals with Parenchymal and Subarachnoid Neurocysticercosis

**DOI:** 10.1371/journal.pntd.0004143

**Published:** 2015-10-27

**Authors:** Iskra Tuero, Sandra Palma, Franco Cabeza, Sarah Saleemi, Silvia Rodriguez, Isidro Gonzales, Holger Mayta, Siddhartha Mahanty, Hector H. Garcia, Robert H. Gilman, Cysticercosis Working Group in Perú

**Affiliations:** 1 Laboratorios de Investigación y Desarrollo, Facultad de Ciencias y Filosofía, Universidad Peruana Cayetano Heredia, Lima, Perú; 2 Asociación Benéfica PRISMA, Lima, Perú; 3 Department of International Health, Bloomberg School of Public Health, Johns Hopkins University, Baltimore, Maryland, United States of America; 4 Johns Hopkins School of Medicine, Baltimore, Maryland, United States of America; 5 Unidad de Cisticercosis, Instituto Nacional de Ciencias Neurológicas, Lima, Perú; 6 Laboratory of Parasitic Diseases, National Institute of Allergy and Infectious Diseases, National Institutes of Health, Bethesda, Maryland, United States of America; University of Würzburg, GERMANY

## Abstract

**Background:**

The ability of *Taenia solium* to modulate the immune system likely contributes to their longevity in the human host. We tested the hypothesis that the nature of the immune response is related to the location of parasite and clinical manifestations of infection.

**Methodology:**

Peripheral blood mononuclear cells (PBMC) were obtained from untreated patients with neurocysticercosis (NCC), categorized as having parenchymal or subarachnoid infection by the presence of cysts exclusively within the parenchyma or in subarachnoid spaces of the brain, and from uninfected (control) individuals matched by age and gender to each patient. Using multiplex detection technology, sera from NCC patients and controls and cytokine production by PBMC after *T*. *solium* antigen (TsAg) stimulation were assayed for levels of inflammatory and regulatory cytokines. PBMC were phenotyped by flow cytometry *ex vivo* and following *in vitro* stimulation with TsAg.

**Principal Findings:**

Sera from patients with parenchymal NCC demonstrated significantly higher Th1 (IFN-γ/IL-12) and Th2 (IL-4/IL-13) cytokine responses and trends towards higher levels of IL-1β/IL-8/IL-5 than those obtained from patients with subarachnoid NCC. Also higher in vitro antigen-driven TNF-β secretion was detected in PBMC supernatants from parenchymal than in subarachnoid NCC. In contrast, there was a significantly higher IL-10 response to TsAg stimulation in patients with subarachnoid NCC compared to parenchymal NCC. Although no differences in regulatory T cells (Tregs) frequencies were found *ex vivo*, there was a trend towards greater expansion of Tregs upon TsAg stimulation in subarachnoid than in parenchymal NCC when data were normalized for the corresponding controls.

**Conclusions/Significance:**

*T*. *solium* infection of the subarachnoid space is associated with an enhanced regulatory immune response compared to infection in the parenchyma. The resulting anti-inflammatory milieu may represent a parasite strategy to maintain a permissive environment in the host or diminish inflammatory damage from the host immune response in the central nervous system.

## Author Summary

Cysticercosis is a parasitic disease caused by the larval cysts of the pork tapeworm *Taenia solium* following accidental ingestion of tapeworm eggs. Neurocysticercosis is a leading cause of seizures and epilepsy in developing countries. The clinical manifestations depend on many factors including the number, size, location, stage of the parasites and host age, gender and immune responses. However, the most important determinant is whether the infection is restricted to the brain parenchyma (associated with seizures disorders but generally with a good prognosis) or if it involves the subarachnoid spaces in the brain (associated with an exuberant growth of the parasite, intracranial hypertension, and often resulting in mortality). In the current study, we characterized the relationship between *T*. *solium* specific immune responses and cyst location. We show that patients with subarachnoid disease have higher suppresive immune responses than the parenchymal group, potentially representing a parasite strategy to survive. Our findings implicate immune responses to be among the mechanisms that underlie the diverse clinical manifestations in neurocysticercosis.

## Introduction

Human cysticercosis involving the central nervous system (CNS), or neurocysticercosis (NCC), is one of the most common neurological infections in developing countries, accounting for as much as a third of adult onset epilepsy in endemic regions [1–3]. The infection results from the accidental ingestion of *Taenia solium* eggs. Following release in the duodenum and activation by digestive enzymes, oncospheres rapidly migrate into the blood and eventually reach the brain and other tissues. In the CNS, oncospheres transform into metacestodes or cysticerci and can survive intact for many years [3,4]. Disease occurs with an initial asymptomatic phase and frequently progresses to a symptomatic phase with a variety of symptoms including headaches, increased intracranial pressure and seizures. Progression to symptomatic NCC is usually associated with larval degeneration resulting from parasite aging, progressive destruction from inflammatory host immune responses over time, or rapidly after anti-helminthic treatment [4].

NCC demonstrates a remarkably heterogeneous presentation with a wide variety of clinical manifestations. The symptoms and signs of infection vary depending on cyst size, viability, and/or location within the brain [3–5]. Previous studies correlating differences in immunological responses to different clinical manifestations of NCC have frequently been inconsistent in the inclusion criteria of cases [6–8]. Studies that investigated the relationship between immune responses and disease manifestations of NCC have generally classified their study populations based on symptomatology [6,9–12]. A significant issue with classifying disease by the presence or absence of symptoms is that individuals can remain symptomatic, even with severe symptoms, well after the death of the parasite [4]. Thus, the study groups included individuals with heterogeneous disease states, hence similarly heterogeneous immune responses [6,8,10,12]. In addition, in most previous studies the patient populations were studied also differed in terms of anthelmintic and corticosteroids treatment [6–8]. Furthermore, a wide variety of parasite antigens have been used to analyze the specific immune response resulting in further heterogeneity in the host immune response. Host factors such as age, gender and the nature of the immune response can also influence the clinical presentation of NCC. Thus, independent of gender, older NCC patients frequently have a predilection for multiples parasite and vesicular cysts. While an increased inflammatory response, reflected by high leukocyte counts in CSF and tomographic evidence of inflammation, is seen more commonly in female NCC patients [13,14].

The prognosis of cerebral cysticercosis generally correlates with the varying duration and severity of the disease that is dependent on whether the cyst location is in parenchymal sites, subarachnoid spaces, or in the major sulci [4]. In the present study, we compared host immune responses in subarachnoid disease, which is characterized by an exuberant or unrestrained growth of the parasite membrane, and parenchymal disease, associated with granulomas and calcification that would presumably sequester the parasite. Further, by matching patients with an uninfected control group by age and gender, we were able to relate the difference between individuals in the two infected subgroups could be compared directly to a reference group, normalizing for within group variability. Thus, the goal of this study was to correlate the *T*. *solium*-specific immune response in neurocysticercosis with disease manifestations as determined by cyst location. We focused on the immune responses in circulating immune cells with the hope that a better understanding of the peripheral immune response may reflect and provide context to local immune responses around the cyst in the CNS.

## Methods

### Study subjects

For this study, we enrolled 32 patients diagnosed with NCC based on criteria specified in the General protocol for Investigation in Neurocysticercosis for the Instituto Nacional de Ciencias Neurológicas, Lima, Peru (protocol # 01034), that included clinical evaluation, neuroimaging, and serological testing. The patients were classified by the location of cysticerci in parenchymal (n = 16) or subarachnoid (n = 13) spaces based on imaging data. Three subjects who had both parenchymal and subarachnoid cysts were excluded from the analysis, resulting in a study size of 29 individuals. To control for exposure to other helminths and immunologically relevant factors, each patient (n = 29) was matched to an uninfected subject by age and gender. The control group consisted of individuals resident in a region non-endemic for cysticercosis, and all were seronegative for cysticercosis and *T*. *solium* taeniasis by the standard diagnostic test, an enzyme-linked immune transfer blot (EITB) [15]; no imaging data were obtained from these uninfected individuals. None of the participants had received steroids or anti-parasitic treatment at the time of blood sampling. Fresh blood specimens were collected from patients and control subjects to obtain mononuclear cells and sera and NCC patients were treated with anthelmintics and anti-inflammatory therapy, if appropriate, in follow up after blood sampling.

### Ethics statement

The study was approved by the Ethics Committee of both the Universidad Peruana Cayetano Heredia and the Instituto Nacional de Ciencias Neurológicas. The Institutional Review Board (IRB) of the Universidad Peruana Cayetano Heredia, Lima, Peru approved the protocol (protocol # 54702) following the principles expressed in the Declaration of Helsinki (1975). Informed consents were signed by all subjects. NCC patients were under continuing care by staff physicians, and received standard of care treatment after sample collection

### Antigen preparation

*T*. *solium* cysticerci were obtained from naturally infected pigs, purchased from cysticercosis-endemic rural regions of Peru. Cysts recovered from pig muscles were washed several times with sterile phosphate buffered saline (PBS), then homogenized and sonicated at 4°C. Cell-free extracts were obtained after centrifugation at 500 g for 10 min. Proteins were quantified using the BCA assay (bicinchoninic acid protein assay; Pierce Biotechnology, Inc., Rockford, IL).

### PBMC isolation and *in vitro* stimulation with *T*. *solium* antigen

Fresh human peripheral blood was obtained and used as a source of PBMC, separated by differential centrifugation using Ficoll-Paque Plus (GE, Piscataway, NJ), according to the manufacturer’s protocol. Cells were set up for *in vitro* culture in 24-well plates at a concentration of 1x10^6^ cells/well (for cell phenotyping) and 96-well plates at 2x10^5^ cells/well (for measurement of cytokine production) in RPMI 1640 medium (Gibco) with 5% heat-inactivated human serum, penicillin and streptomycin, in one of the following conditions: (1) medium alone, (2) *T*. *solium* antigen (TsAg): 20 and 40 μg/ml and (3) *Mycobacterium tuberculosis* purified protein derivative (PPD; 5μg/ml) (Mycos Research) was used as a positive control for measuring an *in vitro* immune response. Culture reagents had minimal lipopolysaccharide (LPS) contamination as assessed by LAL (Limulus amebocyte Lysate test; sensitivity 0.03EU/ml). Cell cultures were incubated at 37°C for 3 days (cell phenotyping) or 48 h (cytokine production).

### Cell phenotyping

The frequency of T cell subpopulations (expressing CD3^+^, CD4^+^ or CD8^+^ cells), NK cells (CD3^-^CD16^+^CD56^+^), B cells (CD19^+^) or regulatory T cells (Tregs: defined as CD4^+^CD25^+^CD127^low/-^Foxp3^+^) were determined for each study subject using standard phenotyping protocols provided by the manufacturers. For this purpose, the following monoclonal antibodies were purchased from Becton Dickinson (San Diego, CA) or eBioscience (San Diego, CA): FITC-conjugated anti-CD3 (clone HIT3a) and anti-CD25 (clone M-A251); PE-conjugated anti-CD16 (clone 3G8) and anti-CD127 (eBioRDR5); PerCP-conjugated anti-CD4 (clone S3.5) and anti-CD19 (clone SJ25-C1); APC-conjugated anti-CD8 (clone RPA-T8), anti-CD56 (clone MEM-188) and anti-FoxP3 (clone 236A/E7).

The cellular phenotype in PBMC was determined by flow cytometry *ex vivo* and following 3 days of culture in medium with or without parasite antigens. After washing, mononuclear cells were stained for specific surface molecules and finally fixed. For FoxP3 intracellular staining, after CD4, CD25 and CD127 surface staining, cells were fixed/permeabilized with FoxP3 staining buffer set (eBioscience) and incubated with the FoxP3 antibody. After 30min of incubation at 4°C cells were washed once with permeabilization/wash buffer and resuspended in 1% paraformaldehyde in PBS. For each sample of PBMC, at least 10,000 target events were acquired on a FACS Calibur (Becton Dickinson) and analyzed using Cellquest software (Becton Dickinson).

### Multiplex analysis for cytokine concentrations

Sera and supernatants from PBMC cultured for 48 h at 37°C with different concentrations of *T*. *solium* antigen were assayed for cytokines: IL-1β, IL-12, interferon gamma (IFN-γ), IL-4, IL-13, IL-10, Granulocyte colony-stimulating factor (G-CSF), vascular endothelial growth factor (VEGF), IL-1rα, IL-2, IL-5, IL-6, IL-7, IL-8, IL-9, IL-15, IL-17, eotaxin, basic FGF, GM-CSF, IP-10, MCP-1, MIP-1α, MIP-1β, PDGF-B, RANTES, and TNF-α using Luminex xMAP technology with a Bio-Plex Human cytokine 27-Plex kit. Cytokine concentrations were expressed as pg/ml using manufacturer-provided standards.

### Statistical analysis

A patient:matched control ratio (r) was calculated for each cytokine concentration and the frequency of each cell sub-population to normalize the patient’s data with the matched control. Statistical significance of differences between patients and controls were tested using a one sample t-test for the difference of ratios from 1.0. The Mann Whitney U rank sum test was used to compare the immune response frequencies of NCC patients with parenchymal vs subarachnoid cyst location. The Wilcoxon signed rank test was used to compare TsAg vs medium for the effect of antigen stimulation. A *P*-value of <0.05 was considered statistically significant. All analyses were corrected for multiple comparisons by the software (GraphPad Prism V6) and the corrected p-values are used in data interpretations.

## Results

### Clinical features of NCC patients

NCC patients were seropositive on EITB and were assigned to the parenchymal or subarachnoid disease groups based on their imaging studies as described by Garcia *et al*.[16]. Patients with parenchymal NCC were included only if they had two or more viable cysts, to minimize the chances of misdiagnosis, and patients with subarachnoid disease were included in this group if they had parasitic lesions only in the basal subarachnoid spaces or in the Sylvian fissure, outside the brain parenchyma. Patients with subarachnoid NCC had parasite membranes and/or cysts identified in the extraparenchymal spaces on MRI imaging, but none had cysts in the brain parenchyma. Three subjects (among the 32 study participants) who had both subarachnoid and parenchymal cysts were excluded for analysis, along with their matched controls. Uninfected control subjects were seronegative on EITB.

All patients with parenchymal cysts (n = 16) had at least two viable brain cysts. Eight of these patients (8/16, 50%) also had additional calcified cysts and one (1/16, 6%) also had a degenerating cyst. However, none of these patients had subarachnoid disease apparent on their brain MRI examinations. In the subarachnoid NCC group (n = 13), five patients (5/13, 38.5%) also had parenchymal calcifications, but none had cysts in the parenchyma (S1 Table).

### Cytokine levels in serum from NCC patients

**Fig 1:**
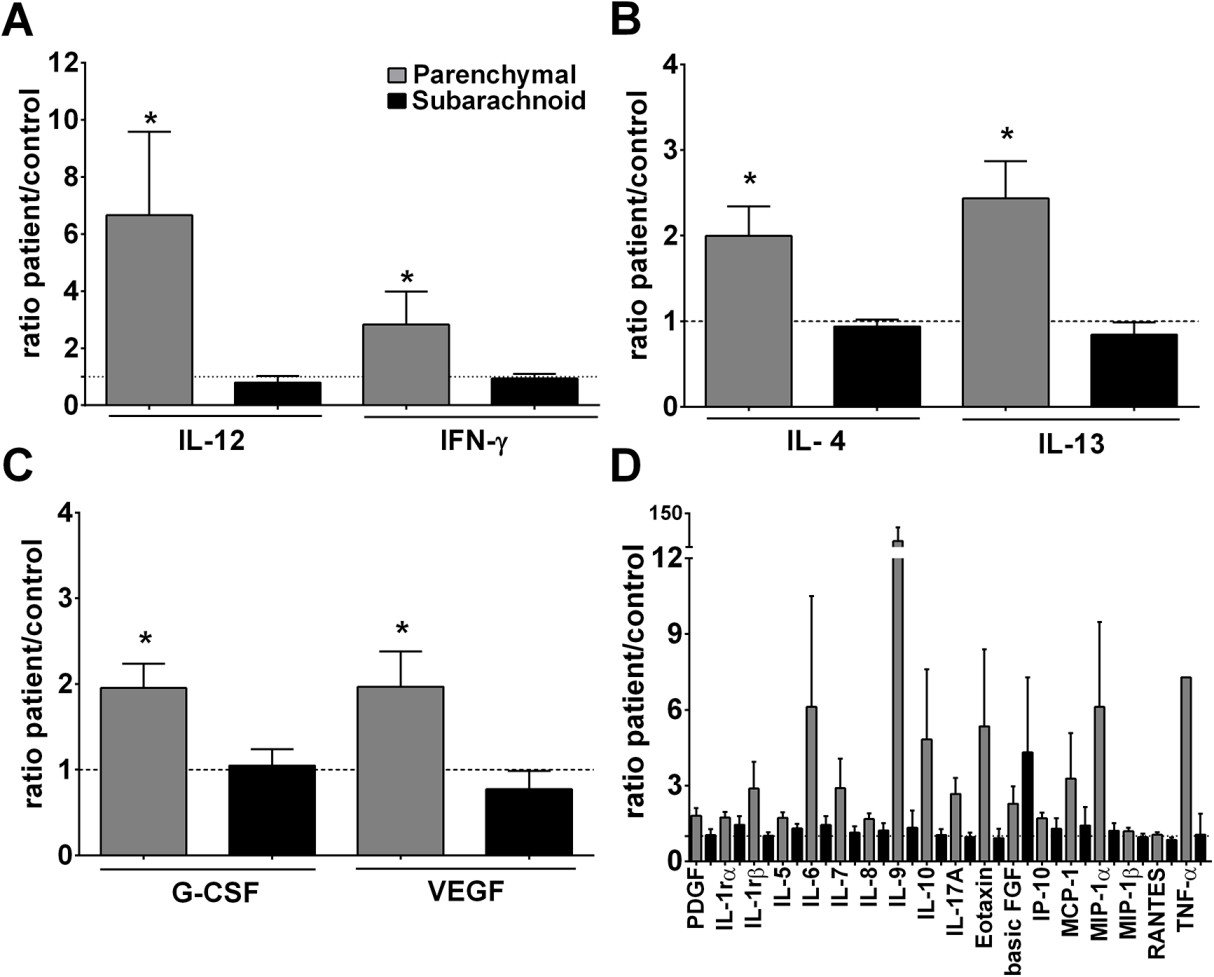
Stronger peripheral pro-inflammatory responses in serum are associated with parenchymal disease. (A) Inflammatory, (B) Th2 cytokines and (C) growth factors and (D) other cytokines/chemokines levels in sera were assessed by multiplex bead array technology. The results are shown as a patient:matched control ratio calculated for each cytokine concentration. Data presented are the mean ± SEM of 16 NCC patients with cysts in parenchymal or 13 patients with subarachnoid locations. * p<0.05 indicates statistically significant differences between the groups compared by using the Mann Whitney U test.

The cytokine concentrations (pg/ml) in NCC patients were normalized to the values in the matched control as a ratio of patient:matched control and the mean values (± standard error of the mean (SEM)) of the parenchymal NCC patients were compared to those of subarachnoid NCC patients (Fig 1). Compared to patients with subarachnoid infections, patients with parenchymal cysts had higher levels of the circulating pro-inflammatory cytokines IL-12 (patient/control ratio (r) = 6.7±2.9 for parenchymal and r = 0.79±0.23 for subarachnoid disease), IFN-γ (patient/control r = 2.8±1.2 for parenchymal and r = 0.94±0.16 for subarachnoid disease; p<0.05, Fig 1A). Strong trends towards higher levels of IL-1β (p = 0.052), IL-8 (p = 0.074) and IL-9 (p = 0.067) were also apparent in parenchymal NCC compared to subarachnoid NCC (Fig 1D). Individuals with parenchymal cysts also showed higher Th2 cytokines: IL-4 (patient/control r = 2.0±0.3 for parenchymal and r = 0.94±0.08 for subarachnoid disease) and IL-13 (patient/control r = 2.4±0.4; for parenchymal and r = 0.84±0.14 for subarachnoid disease p<0.05; Fig 1B), and higher levels of the growth factors G-CSF (patient/control r = 2.0±0.3 for parenchymal and r = 1.05±0.19 for subarachnoid disease) and VEGF (patient/control r = 2.0±0.4; for parenchymal and r = 0.77±0.21 for subarachnoid disease; p<0.05, Fig 1C). Similar trends towards higher levels of IL-5 (p = 0.66) and eotaxin (p = 0.096) were observed in parenchymal vs. subarachnoid NCC (Fig 1D). No significant differences were noted between the two groups for PDGF-B, IL-1rα, IL-6, IL-7, IL-10, IL-17A, basic FGF, IP-10, MCP-1, MIP-1α, MIP-1β, RANTES, and TNF-α in the serum. No IL-2, IL-15 and GM-CSF were detected in sera from any of the study groups (Fig 1D). In contrast to the normalized cytokine data discussed above, when cytokines in serum expressed as pg/ml were compared between the NCC patients and controls, patients with subarachnoid disease show higher levels of IL-1α, IL-8 (S1A Fig; p<0.05) and IL-5 (S1B Fig; p<0.05) than patients with parenchymal NCC and controls subjects, whereas MCP-1, MIP-1α, RANTES (S1C Fig), IL-4 (S1B Fig), PDGF, G-CSF (S1D Fig) were higher (p<0.05) in subarachnoid patients than in the parenchymal group.

### *T*. *solium* antigen-stimulated cytokine production by PBMC

The optimal antigen concentration for PBMC stimulation was determined using fresh PBMC from subjects without NCC to select a dose range with low non-specific stimulatory effects. For *in vitro* cell proliferation (measured by thymidine incorporation into DNA), 20 to 40 μg/ml of TsAg was determined to be optimal with the best stimulation indices (SIs) at 6 days (SI at 20 μg/ml: 6.8±2.1 and 40 μg/ml: 12.5±5.1) (S2 Fig). Subsequently, these TsAg concentrations were employed for i*n vitro* stimulation of PBMC from patients with NCC for comparison of antigen-driven cytokine secretion between patients with parenchymal and subarachnoid disease.

**Fig 2:**
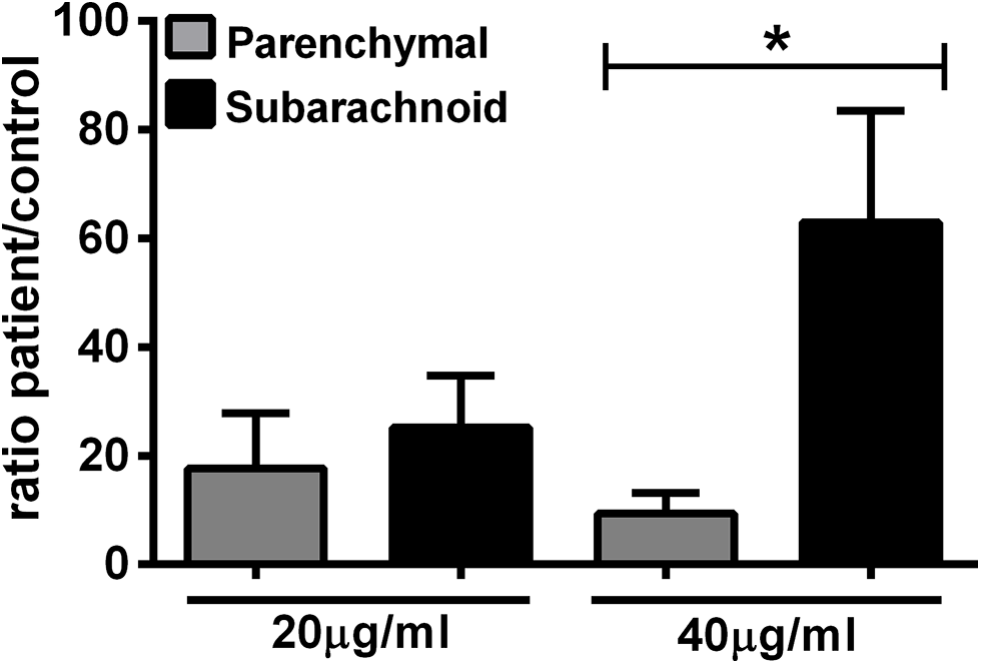
Regulatory immune responses to parasite antigens are higher in subarachnoid disease compared to parenchymal disease. Culture supernatant were collected at 48 h and assessed for IL-10 production by PBMC after stimulation with *T*. *solium* antigen (20 and 40 μg/ml). The results are shown as a patient:matched control ratio calculated for cytokine concentrations. Data presented are the mean ± SEM of 16 NCC patients with parenchymal or 13 patients with subarachnoid location, respectively. *p<0.05 indicate statistically significant differences between the groups compared by using Mann Whitney U test.

To assess if TsAg-induced cytokine production *in vitro* differed between the two clinical forms of NCC, we evaluated the cytokine levels in supernatants of PBMC cultures from parenchymal and subarachnoid patient groups, normalized for their matched controls, following *in vitro* stimulation with parasite antigen. We tested a total of 27 cytokines and found that only IL-10 values differed significantly between the two groups of patients (S1 Table and Fig 2). After 48h of Ag stimulation, when parenchymal and subarachnoid group were compared for IL-10 production, PBMC from the subarachnoid group elicited a higher IL-10 production (patient/control r = 62.99±20.6) in response to TsAg (40 μg/ml) than the parenchymal group (patient/control r = 9.4±3.8; p<0.05; Fig 2). There were strong trends towards higher levels of TNF-α (p = 0.051) and lower levels of IL-5 (p = 0.075) in parenchymal NCC compared to subarachnoid NCC (S2 Table). In contrast to the normalized data, when the cytokines levels expressed in pg/ml were directly compared between the study groups (S3 Fig), PBMC from patients with parenchymal disease TsAg produced higher levels of TsAg-driven IL-2, IL-4, IL-5, IL-8, IL-10, IL-12, IL-13, MCP-1, MIP-1β and TNF-α than patients with subarachnoid disease and control subjects (S3 Fig; p<0.05).

### Changes in lymphocyte subpopulations in response to *T*. *solium* antigens

**Fig 3:**
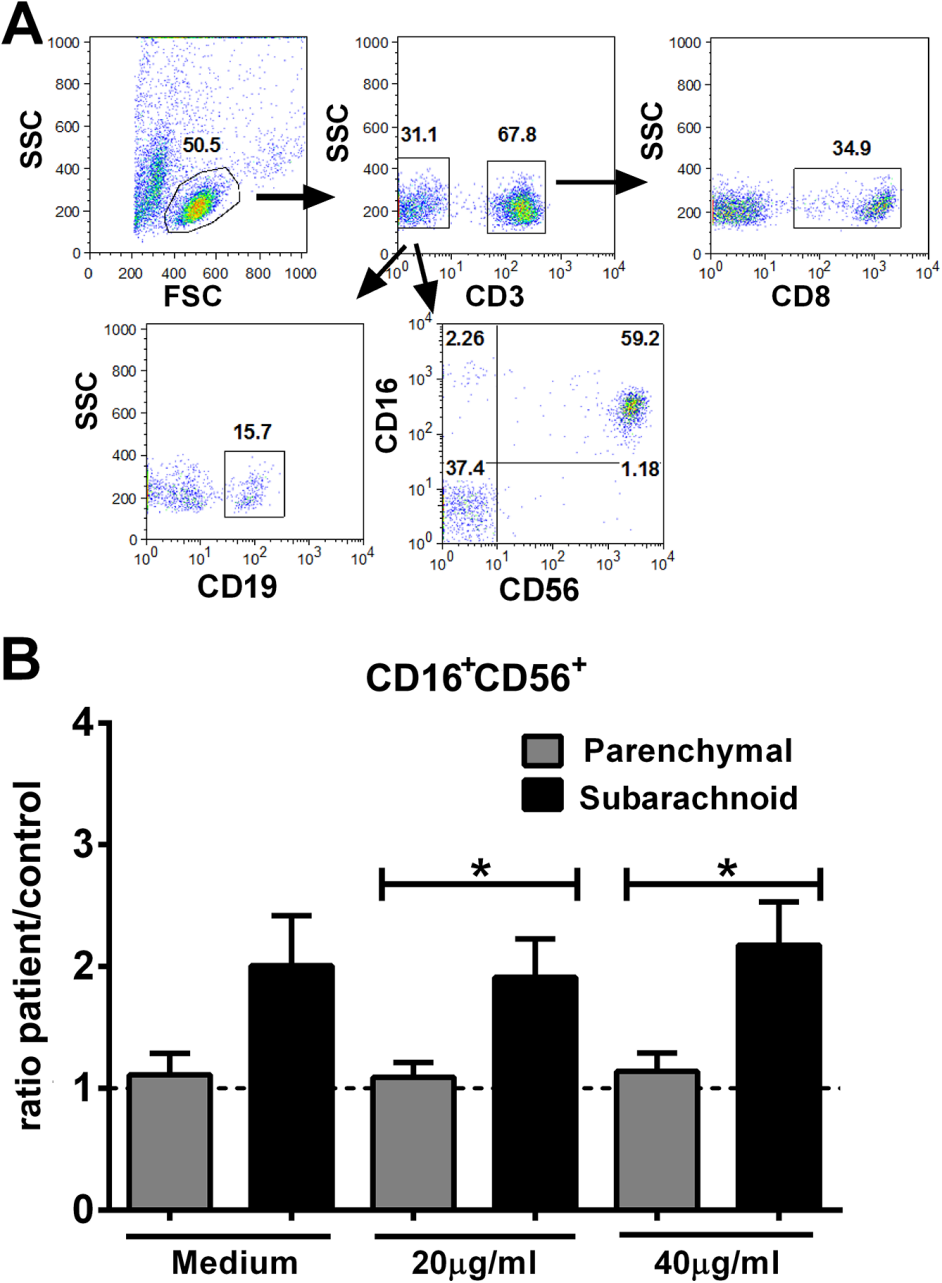
NCC patients with subarachnoid cysts show expanded peripheral CD16^+^CD56^+^ cells. (A) Representative FACS staining of lymphocytes in peripheral blood from a healthy subject. T cells, NK and B cells were identified by gating on the lymphocyte population using forward and side scatter parameters (B) Phenotyping of PBMC for NK cells (CD16^+^CD56^+^) was performed by flow cytometry. Data presented are the mean ± SEM of the ratio of cell frequencies (%) for patient:matched controls for NCC patients with parenchymal (n = 16) and subarachnoid (n = 13) cysts. * indicate statistically significant differences (p<0.05) between the three groups compared pairwise with medium alone by Mann Whitney U test.

We phenotyped a number of lymphocyte subsets in order to identify the PBMC population that may have expanded in response to *T*. *solium* exposure and may, thus, have roles in regulating inflammatory responses in the two NCC study groups, with either parenchymal or subarachnoid NCC. These data were also normalized for matched controls as we did for cytokine data in serum and supernatants (above), to eliminate the influence of age and gender. The cell subpopulations enumerated included T cells, B cells and NK cells (CD16^+^CD56^+^) (Fig 3A). The only lymphocyte subpopulation that was found to differ significantly between the parenchymal NCC and subarachnoid NCC groups was the CD16/CD56 positive (NK) subset, indicating an expansion of these cells in the latter group (Fig 3B). As with the differences in cytokine levels in serum and supernatants of PBMC, we found that differences in frequency data for cell phenotypes between parenchymal and subarachnoid NCC were altered when analyzed after normalization with corresponding controls (See S4 and S5 Figs), suggesting that age and gender affected the expression of these parameters.

**Fig 4:**
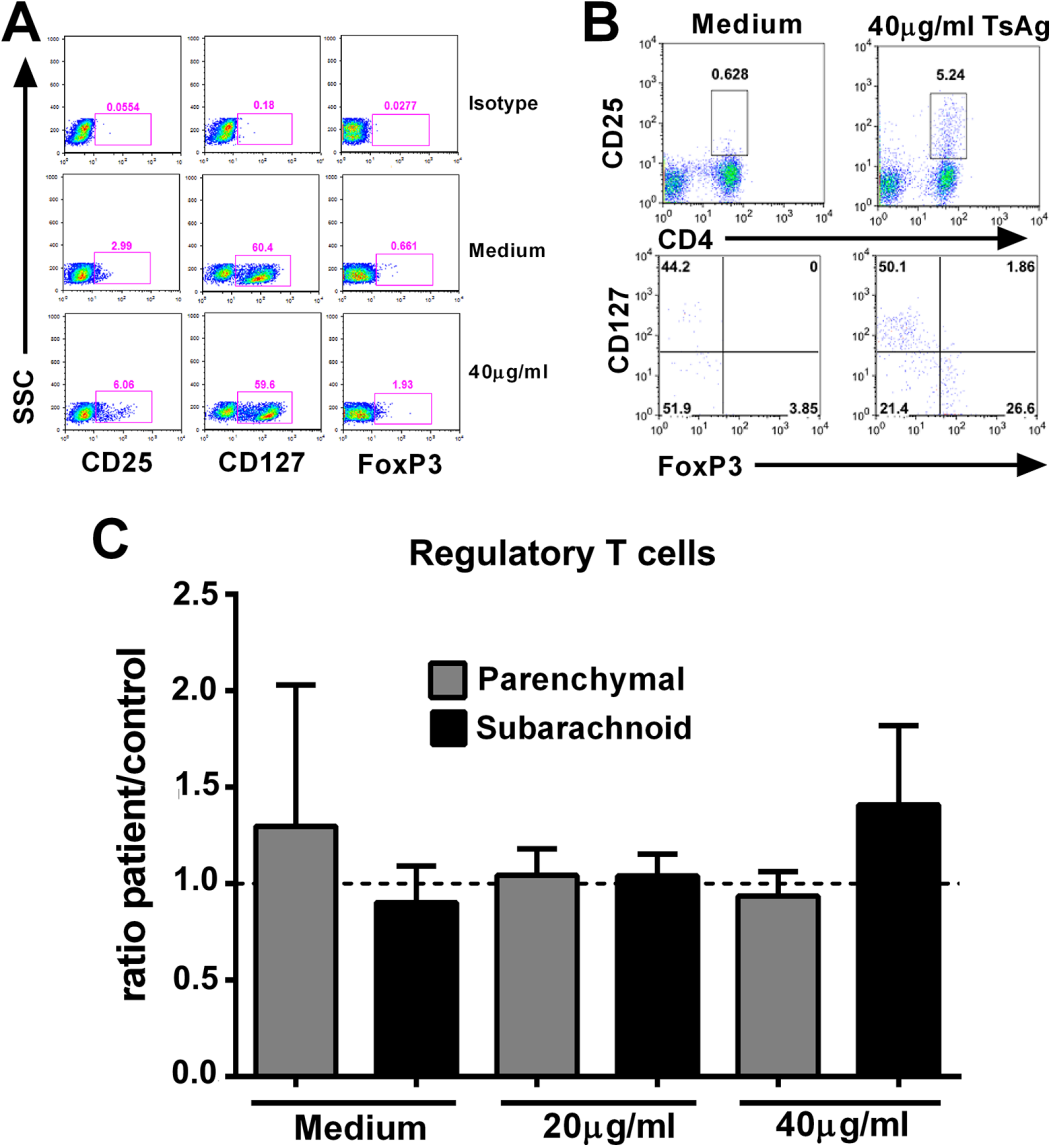
Frequencies of circulating regulatory T-cells in PBMC following *T*. *solium* antigen stimulation. (A) Representative plots showing fluorescence for the isotype control mAbs for Tregs-specific mAbs in lymphocytes, and expression of CD25, CD127 and FoxP3 in unstimulated and TS Ag-stimulated cells (B) Gating strategy for Treg analysis in PBMC from NCC patient. Cells defined by the lymphocytes gate were analyzed for CD4 and CD25 expression, and CD4/CD25 double positive cells were analyzed for expression of CD127 and FoxP3 to identify Treg (CD127^low/-^FoxP3^+^) cells and (C) Regulatory T cells in each study group enumerated by flow cytometry after culture with medium alone or *T*. *solium* antigen (20 and 40 μg/ml) on day 3. Data presented are the mean ± SEM of NCC patients with parenchymal (n = 16) and subarachnoid disease (n = 13). * indicate statistically significant differences (p<0.05) between the three groups compared pairwise with medium alone by Mann Whitney U test.

When the frequencies of Treg cells, normalized for gender and age, were compared (Fig 4), differences between subjects with parenchymal and subarachnoid NCC *ex vivo* (S4A Fig) or after TsAg stimulation did not achieve statistical significance (Fig 4C). However, there was a strong trend towards higher frequencies of CD4 cells *ex vivo* (p = 0.057; S4B Fig) and Treg cells (p = 0.051; S5F Fig) in parenchymal NCC compared to subarachnoid disease. Taken together, these data suggest that parenchymal NCC is associated with a predominat pro-inflammatory response. Further, *in vitro* stimulation with parasite antigens led to an expansion of Treg cells that, perhaps due to the small sample size, did not achieve statistical significance. Notably, in the case of antigen-stimulated responses, normalization with matched control subjects did not change the conclusions from the non-normalized frequency data (Figs 4C and S5F).

## Discussion

Cerebral cysticercosis is a remarkably complex and heterogeneous infection that results in a wide spectrum of clinical manifestations and a variable prognosis. A major determinant of the severity of NCC and the nature of it’s clinical manifestations is the parasite location: parenchymal cysts are frequently associated with seizures but generally have a better outcome following treatment than does extraparenchymal disease, which is commonly associated with serious neurological and vascular complications such as intracranial hypertension, mass effects, strokes and frequently, with fatality [3–5,12]. The present study was designed to investigate the relationship between the *T*. *solium* specific-immune responses and disease manifestations, with a goal of understanding the mechanisms underlying clinical heterogeneity in NCC. Based largely on the prominent symptomatology and inflammatory reaction in the CSF in subarachnoid NCC (compared to parenchymal cysts) we hypothesized that subarachnoid NCC would be associated with a pro-inflammatory and regulatory immunological state compared to parenchymal NCC. This was a possible consequence of encapsulation of the parasite in parenchymal disease by the inflammatory capsule and sequestration from an activated immune system [11]. However, our findings did not support our proposed model of immune responses in the two forms of NCC.

Contrary to our *a priori* hypothesis the major, and unexpected, finding in our study was that subarachnoid NCC was associated with a less inflammatory and stronger regulatory immune response to parasite antigens. The key observation was that circulating cytokine levels indicated a higher pro-inflammatory status in parenchymal disease, reflected in IL-12 and IFN-γ levels (Fig 1A), compared to subarachnoid disease. Interestingly, this was also accompanied by higher levels of IL-4, IL-13 and growth factors GM-CSF and VEGF (Fig 1B and 1C) suggesting a global immune activation. This was unexpected because the high parasite burden and exuberant growth of the parasite in subarachnoid disease suggested, to us, a stronger inflammatory response than the constrained growth of the parasite in parenchymal locations, in what is considered an immunologically privileged organ. However, patients with subarachnoid cysts demonstrated a systemic cytokine profile similar to that of the control subjects (as shown the patient/control ratio ≤1) with lower levels of circulating pro-inflammatory mediators, circulating Th2 (IL-4 and IL-13; Fig 1B) and higher levels of TsAg-driven IL-10 *in vitro* than those with parenchymal cysts (Fig 2). Of note, higher levels of inflammation in pericystic tissues surrounding parenchymal cysts than around meningeal cysts have also been observed in a pig model of neurocysticercosis, when naturally infected pigs were treated with praziquantel triggering an acute host response (Cangalaya *et al*., submitted for publication), and in experimental intracerebral infections with *T*. *solium* in rats [17].

The prominent regulatory environment we observed in subarachnoid NCC may result from one or more immunological mechanisms. One explanation could be that in subarachnoid disease, because of greater parasite growth in the unconstrained subarachnoid space, a higher local antigen load than occurs in the smaller, constrained cysts located in the parenchyma. In this setting, a higher IL-10 levels can be induced, as reported by Chavarria *et al*. [11] in CSF from patients with subarachnoid NCC disease with severe symptoms. It is also possible that greater access to the parasite antigens in the subarachnoid location (compared to parenchymal cysts) may promote a stronger or preferentially immunosuppressive response in extraparenchymal sites, either directly through parasite-derived molecules, or indirectly through manipulation of the host immune system. This mechanism could explain the relative downregulation of inflammation in subarachnoid NCC, which is associated with high parasite burdens in locations accessible to the immune effector cells, whereas in parenchymal disease, encapsulation of the parasite and the consequent sequestration of parasite-derived molecules may inhibit the downregulatory stimulus that potentially protects the parasite. However, in the current cross-sectional study we were unable to directly test this hypothesis.

Some of our observations differ from reports from other investigators who have characterized the immune responses to parasite Ags in NCC patients. Recently, the observation of expanded Tregs subpopulations in NCC patients and after *in vitro* stimulation with *T*. *solium* antigens, has lent support to the notion that Tregs may help in limiting the inflammatory response [10,18–21]. The current, carefully controlled study showed a strong trend towards higher frequencies of Treg cells in subarachnoid NCC compared to parencehymal disease after *in vitro* stimulation (p = 0.051; S5F Fig). Unfortunately, our analysis does not allow us to determine functional differences in the Treg cell populations between the study populations. Functional analysis of these cells or other cellular sources of regulatory cytokines (such as macrophages and Th2 cells) might help to explain their role in the generating the stronger regulatory state associated with subarachnoid NCC. Additionally, our finding of an enhanced regulatory profile in subarachnoid disease is also in contrast to previous studies that reported an increased inflammatory cytokine response in the subarachnoid space, using CSF, compared to the parenchyma [11,12,21]. We believe that the homogeneous clinical features, careful matching with controls for age and gender and absence of treatment in our group of patients support the validity of our observations and may explain the differences found in the immune profile in this study compared to previous reports.

A remarkable aspect about the suppressive or regulatory immune response observed in the subarachnoid NCC patients in our study was that a high IL-10 production in SA patients was seen mainly after stimulation with high concentrations of TsAg (40 μg/ml; Fig 2); this suggests that strong suppressor activity may be restricted to the proximity of cyst where a levels of released parasite antigens are high. Evidence for immunomodulation by parasite antigen that induced CD4^+^CD25^+^FoxP3^+^ Tregs to produce regulatory cytokines such as IL-10 has been reported with *Schistosoma mansoni* antigens [22] and also in infections caused by *Echinococcus* [23,24] and *T*. *solium* [10,18].

We analyzed fresh blood/serum samples from well-defined patients at presentation to a clinical center, before they had initiated steroid or antiparasitic treatment to minimize within-group heterogeneity in disease manifestations, a factor that has compromised some previous studies that relied on symptomatology for disease classification. Disease categories in this study were based strictly on the location of the cysticerci (in the parenchymal or subarachnoid space), defining two populations, one with parenchymal disease alone, and a second with subarachnoid disease without parenchymal cysts. In addition the patients were matched by age and gender to a control group, making for more robust comparisons by controlling for immunologically mediated environmental factors and for other helminth infections. We believe that this approach allowed us to have well-controlled and clinically uniform patient groups.

Although we limited our analysis of cell populations to circulating immune cells, there is good reason to expect that the differences in circulating cell subpopulations between the study groups likely reflect those in the CSF compartment, particularly in subarachnoid infections. This has been reported in a previous study comparing blood and CSF cell phenotype from NCC patients [10]. The blood brain barrier has been shown to be compromised around degenerating cysts which would allow two-way trafficking of cells between the circulation and the CNS [25]. Furthermore, in clinical settings and future investigations, blood samples are vastly more accessible than CSF samples from patients, and not technically limited by small numbers of cells present in CSF.

The systemic immune responses, as seen from the cytokine profile analysis of sera from NCC patients with cysts in the parenchyma, elicits not only a broad repertoire of inflammatory cytokines and Th2 responses but also of growth factors (G-CSF and VEGF) (Fig 1C). During cyst development, the survival and growth of the parasite is dependent on vascularization and angiogenesis, and an increased production of VEGF could be beneficial for this process by promoting the development of capsule that functionally isolates the parasite, and may protect it from interactions with damaging responses [26].

Although the analysis of cytokine levels and responses to TsAg stimulation show that patients with parenchymal NCC disease exhibited a higher systemic inflammatory profile compared to subarachnoid NCC disease, few differences were observed in the frequencies of a number of lymphocyte subpopulations *ex vivo*, with or without normalization with matched controls. The lack of differences between parenchymal and subarachnoid NCC in the phenotypic analysis does not rule out functional differences between lymphocyte subpopulations in the two groups. In this regard, we did find that Ag stimulation induced a greater expansion of NK cells in the subarachnoid NCC patients than in the parenchymal patients (Fig 3B). This expansion may represent a reaction to the downregulatory stimulus to T cells associated with the parasite in subarachnoid locations, although the NK cells role in inflammatory neurological disorders is still unclear [27]. Interestingly, differences in the outcome of comparisons when data were normalized or not normalized for matched controls strongly suggest that matching of patients and control groups is important and appropriate in this disease, and that the age and gender influence the immune response to this parasite significantly.

The marked differences in pathophysiology between parenchymal and subarachnoid infections, with chronic inflammation and progressive disease being very frequent in subarachnoid cases, suggested to us that subarachnoid disease would induce a more pro-inflammatory response than would parenchymal disease. Our data shows that the location of infection influences the nature of the peripheral immune response, and that unexpectedly and perhaps counter-intuitively, subarachnoid disease, the form that presents clinically with inflammatory pathology in the CNS has a more regulatory immune environment than parenchymal disease. In this type of cross-sectional study design it is not possible to determine if the regulatory state in subarachnoid disease is the consequence of an anti-inflammatory (or regulatory) response to a stronger inflammatory response than that seen in parenchymal NCC. Understanding the immune pathogenesis of NCC might in the future lead to more specific treatment by modeling the immune response.

### Members of the Cysticercosis Working Group in Perú

Armando E. Gonzalez, DVM, PhD; Victor C.W.Tsang, PhD (Coordination Board); Herbert Saavedra, MD; Manuel Martinez, MD; Manuel Alvarado, MD (Instituto Nacional de Ciencias Neurológicas, Lima, Perú); Manuela Verastegui, PhD; Mirko Zimic, PhD; Javier Bustos, MD, MPH; Cristina Guerra, PhD; Yesenia Castillo, MSc; Yagahira Castro, MSc (Universidad Peruana Cayetano Heredia, Lima, Perú); Maria T. Lopez, DVM, PhD; Cesar M. Gavidia, DVM, PhD (School of Veterinary Medicine, Universidad Nacional Mayor de San Marcos, Lima, Perú); Luz M. Moyano, MD; Viterbo Ayvar, DVM (Cysticercosis Elimination Program, Tumbes, Perú); Theodore E. Nash, MD; John Noh, BS, Sukwan Handali, MD (CDC, Atlanta, GA); Jon Friedland (Imperial College, London, UK).

## Supporting Information

S1 Fig Cytokine profiles in sera from NCC patients and controls. (A) Inflammatory cytokines, (B) Th2 cytokines, (C) inflammatory chemokines and (D) growth factors, assessed by multiplex bead array analysis. Data presented are the mean ± SEM of patients with parenchymal (n = 16) or subarachnoid NCC (n = 13) or controls (n = 29), respectively.
(TIF)

S2 Fig *T*. *solium* antigen induced proliferation of PBMC from uninfected subjects. PBMC were cultured in microtiter wells at 2x10^5^/well with one of the following: 1) medium alone, or 2) TsAg (80, 40, 20, 5 or 2.5 μg/ml). Cellular proliferation was assessed at 6 days by measuring the amount of (^3^H) thymidine incorporation. Results are the mean of 7 different control subjects and are expressed as proliferation index, which is the ratio of the counts per minute (CPM) of cultures exposed to antigen to CPM from cultures treated with medium alone.
(TIF)

S3 Fig Cytokine production by PBMC from NCC patients and controls following *in vitro T*. *solium* antigen stimulation. Supernatant was analyzed after culture in medium alone, PPD (5μg/ml) or *T*. *solium* antigen (20 or 40 μg/ml) for 48h. Results presented were normalized by subtraction of corresponding cytokine vales in medium alone. Data presented are the mean ± SEM of patients with parenchymal (n = 16) or subarachnoid NCC (n = 13), and controls (n = 29).
(TIF)

S4 Fig Frequencies of lymphocyte subpopulations normalized with matched controls. The frequencies of lymphocytes in PBMC from NCC patients was normalized against age and gender matched uninfected controls. Lymphocyte populations were analyzed for frequencies of CD4^+^ T cells (B), CD8^+^ T cells (C) and B cells (CD19^+^) in fresh samples (*ex vivo*) (A) and after *in vitro* culture in medium alone, 20 or 40 μg/ml *T*. *solium* antigen for 3 days (B-D). Data presented are the mean ± SEM for patients with parenchymal (n = 16) or subarachnoid (n = 13), respectively.
(TIF)

S5 Fig Frequencies of lymphocytes in patients and controls. (A) *Ex vixo* frequency analysis of PBMC for indicated lymphocyte subpopulations in the three study groups (parenchymal [grey], subarachnoid NCC [black] and the control group [unshaded]; (B) Frequencies of CD4^+^ T cells, (C) CD19^+^ cells, (D) CD8^+^ T cells, CD16^+^/CD56^+^ (NK cells; E) and Treg cells (F) after *in vitro* culture with 20 and 40 μg/ml TsAg for 3 days. Data presented are the mean ± SEM of patients with parenchymal (n = 16) or subarachnoid NCC (n = 13), respectively.
(TIF)

S1 Table Clinical and radiological characteristics of patients with neurocysticercosis. (DOCX)

S2 Table Cytokine production by PBMC from NCC patients following stimulation with *T*. *solium* antigen. (DOCX)
