# Supplementary figures and images for: A Comparative Study of Peripheral Immune Responses to Taenia solium in Individuals with Parenchymal and Subarachnoid Neurocysticercosis
Source: PLoS Negl Trop Dis. 2015 Oct 27;9(10):e0004143. doi: 10.1371/journal.pntd.0004143 (PMC4624727; doi:10.1371/journal.pntd.0004143)

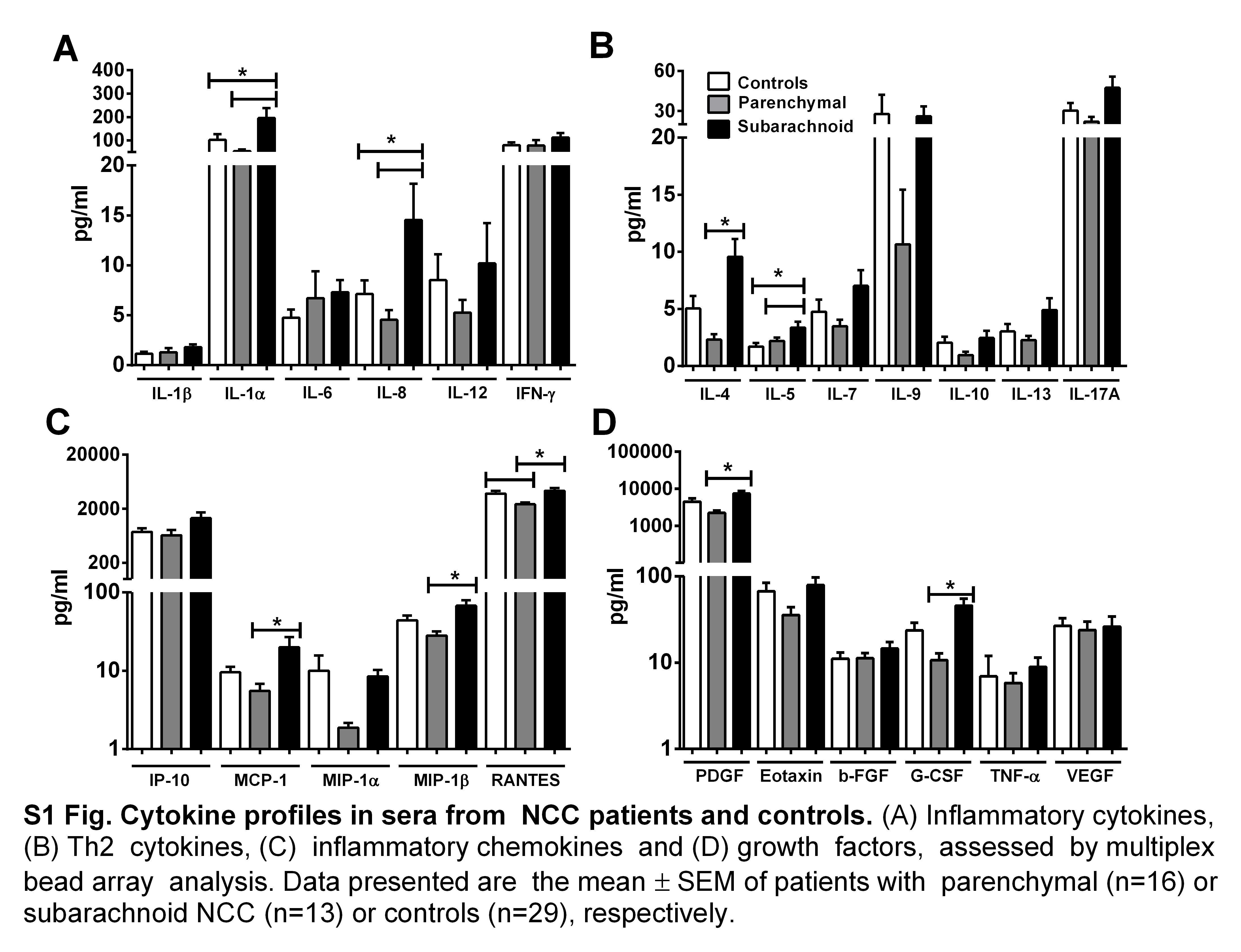

Supplement: S1 Fig — (A) Inflammatory cytokines, (B) Th2 cytokines, (C) inflammatory chemokines and (D) growth factors, assessed by multiplex bead array analysis. Data presented are the mean ± SEM of patients with parenchymal (n = 16) or subarachnoid NCC (n = 13) or controls (n = 29), respectively. (TIF) [file pntd.0004143.s001.tif]

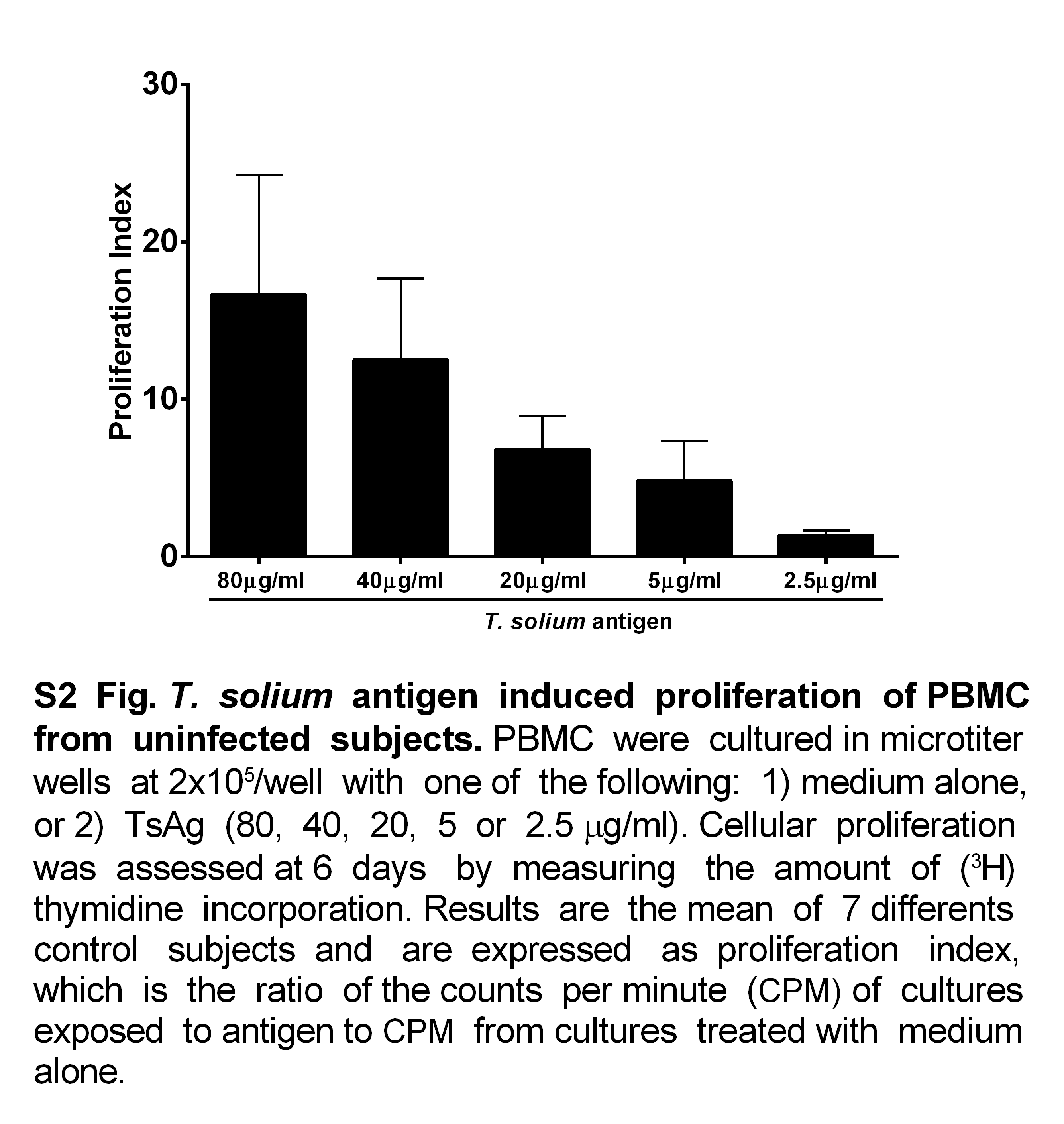

Supplement: S2 Fig — PBMC were cultured in microtiter wells at 2x10^5^/well with one of the following: 1) medium alone, or 2) TsAg (80, 40, 20, 5 or 2.5 μg/ml). Cellular proliferation was assessed at 6 days by measuring the amount of (3H) thymidine incorporation. Results are the mean of 7 different control subjects and are expressed as proliferation index, which is the ratio of the counts per minute (CPM) of cultures exposed to antigen to CPM from cultures treated with medium alone. (TIF) [file pntd.0004143.s002.tif]
