# Supplementary material for: A Comparative Study of Peripheral Immune Responses to Taenia solium in Individuals with Parenchymal and Subarachnoid Neurocysticercosis
Source: PLoS Negl Trop Dis. 2015 Oct 27;9(10):e0004143. doi: 10.1371/journal.pntd.0004143 (PMC4624727; doi:10.1371/journal.pntd.0004143)
Supplement: S1 Table — (DOCX) [file pntd.0004143.s006.docx]

**Supplemental Table 1.** Clinical and radiological characteristics of patients with neurocysticercosis

|  | Patient group (n) | | |
| --- | --- | --- | --- |
|  | Parenchymal  (16) | Subarachnoid  (13) | Controls  (29) |
| Age range (years) † | 28 [22-34] | 38 [28-60] | 29 [24-54] |
| Gender M/F* | 9/7 | 6/7 | 14/15 |
| NCC characteristics |  |  |  |
| Parasitic lesions in subarachnoid spaces | - | 16 |  |
| Viable parenchymal cysts | 16 | - | - |
| Degenerating parenchymal cysts | 1 | - | - |
| Parenchymal brain calcifications | 8 | 5 | - |
|  |  |  |  |

n= number of patients, †Median [interquartile range], *M= male, F= female
