# Supplementary material for: A Comparative Study of Peripheral Immune Responses to Taenia solium in Individuals with Parenchymal and Subarachnoid Neurocysticercosis
Source: PLoS Negl Trop Dis. 2015 Oct 27;9(10):e0004143. doi: 10.1371/journal.pntd.0004143 (PMC4624727; doi:10.1371/journal.pntd.0004143)
Supplement: S2 Table — (DOCX) [file pntd.0004143.s007.docx]

**Supplemental Table S2.** Cytokine production by PBMC from NCC patients after *T. solium* antigen stimulation.

|  | Parenchymal | | Subarachnoid | |
| --- | --- | --- | --- | --- |
|  | 20 μg/ml | 40 μg/ml | 20 μg/ml | 40 μg/ml |
| IL-1b | 0.76 ± 0.36 | 2.10 ± 1.25 | 5.1 ± 4.05 | 5.11 ± 2.37 |
| IL-1a | 6.54 ± 3.98 | 1.92 ± 0.72 | 6.42 ± 3.51 | 2.39 ± 1.23 |
| IL-2 | 2.17 ± 0.44 | 2.18 ± 0.36 | 5.62 ± 2.67 | 2.26 ± 0.61 |
| IL-4 | 19.72 ± 9.77 | 9.52 ± 6.24 | 1.82 ± 0.65 | 3.09 ± 0.99 |
| IL-5 | 7.11 ± 2.29 | 7.69 ± 2.53 | 26.32 ± 10.72 | 47.63 ± 16.93* |
| IL-6 | 1.39 ± 1.07 | 0.99 ± 0.36 | 2.26 ± 0.83 | 8.35 ± 5.05 |
| IL-7 | 0.63 ± 0.53 | 0.71 ± 0.31 | 1.12 ± 1.0 | 2.51 ± 2.31 |
| IL-8 | 1.32 ± 0.45 | 216.6 ± 215.6 | 1.7 ± 1.21 | 0.99 ± 0.32 |
| IL-9 | 0.07 ± 0.07 | 0.84 ± 0.84 | 0.75 ± 0.75 | 3.73 ± 1.19 |
| IL-12 | 8.93 ± 7.35 | 1.46 ± 0.46 | 0.00 | 0.00 |
| IL-13 | 13.23 ± 7.45 | 11.6 ± 3.21 | 5.08 ± 1.55 | 90.86 ± 48.04 |
| IL-15 | 2.43 ± 1.43 | 4.27 ± 2.31 | 4.75 ± 4 | 2.67 ± 1.09 |
| IL-17A | 1.13 ± 0.58 | 1.11 ± 0.55 | 1.02 ± 0.34 | 2.13 ± 0.83 |
| Eotaxin | 0.54 ± 0.39 | 0.27 ± 0.17 | 2.22 ± 0.97 | 0.67 ± 0.29 |
| FGF basic | 1.02 ± 0.63 | 0.22 ± 0.22 | 0.55 ± 0.32 | 1.02 ± 0.37 |
| G-CSF | 1.59 ± 1.00 | 0.98 ± 0.27 | 0.47 ± 0.41 | 1.27 ± 0.63 |
| GM-CSF | 0.85 ± 0.68 | 1.02 ± 1.02 | 0.00 | 0.43 ± 0.0 |
| IFN-g | 1.57 ± 0.85 | 1.11 ± 0.39 | 0.21 ± 0.14 | 2.52 ± 0.77 |
| IP-10 | 0.45 ± 0.23 | 1.25 ± 0.86 | 1.45 ± 1.24 | 2.01 ± 1.66 |
| MCP-1 | 1.94 ± 0.87 | 10.61 ± 8.03 | 4.77 ± 2.76 | 3.61 ± 2.29 |
| MIP-1a | 308.5 ± 307.1 | 4.61 ± 2.15 | 2.29 ± 0.89 | 5.51 ± 4.36 |
| MIP-1b | 3.18 ± 1.96 | 2.79 ± 1.26 | 2.72 ± 1.06 | 1.87 ± 0.62 |
| PDGF-BB | 0.581 ± 0.58 | 0.11 ± 0.11 | 0.00 | 0.00 |
| RANTES | 3.54 ± 2.4 | 1.78 ± 1.03 | 7.46 ± 5.33 | 15.65 ± 14.63 |
| TNF-a | 2.00 ± 0.68 | 3.96 ± 2.59 | 0.2 ± 0.11 | 2.62 ± 1.14 |
| VEGF | 0.12 ± 0.11 | 0.23 ± 0.13 | 0.00 | 0.00 |

Results are expressed as mean ratio (± SEM) of patient:control cytokine levels.

*p<0.05 (20 μg/ml vs 40 μg/ml)
